# A Soluble Immune Effector Binds Both Fungi and Bacteria via Separate Functional Domains

**DOI:** 10.3389/fimmu.2019.00369

**Published:** 2019-03-06

**Authors:** Assunta Liberti, John P. Cannon, Gary W. Litman, Larry J. Dishaw

**Affiliations:** ^1^Department of Pediatrics, Morsani College of Medicine, University of South Florida, Tampa, FL, United States; ^2^Department of Molecular Genetics, Children's Research Institute, Johns Hopkins All Children's Hospital, St. Petersburg, FL, United States

**Keywords:** VCBP-C, *Ciona*, fungal-immune interaction, mycobiota, innate immunity, gut immunity, transkingdom interactions, host-microorganism interactions

## Abstract

The gut microbiome of animals consists of diverse microorganisms that include both prokaryotes and eukaryotes. Complex interactions occur among these inhabitants, as well as with the immune system of the host, and profoundly influence the overall health of both the host and its microbial symbionts. Despite the enormous importance for the host to regulate its gut microbiome, the extent to which animals generate immune-related molecules with the capacity to directly influence polymicrobial interactions remains unclear. The urochordate, *Ciona robusta*, is a model organism that has been adapted to experimental studies of host/microbiome interactions. *Ciona* variable-region containing chitin-binding proteins (VCBPs) are innate immune effectors, composed of immunoglobulin (Ig) variable regions and a chitin-binding domain (CBD) and are expressed in high abundance in the gut. It was previously shown that VCBP-C binds bacteria and influences both phagocytosis by granular amoebocytes and biofilm formation via its Ig domains. We show here that the CBD of VCBP-C independently recognizes chitin molecules present in the cell walls, sporangia (spore-forming bodies), and spores of a diverse set of filamentous fungi isolated from the gut of *Ciona*. To our knowledge, this is the first description of a secreted Ig-containing immune molecule with the capacity to directly promote transkingdom interactions through simultaneous binding by independent structural domains and could have broad implications in modulating the establishment, succession, and homeostasis of gut microbiomes.

## Introduction

The gut microbiota consists of diverse communities of microorganisms, including: bacteria, archaea, viruses, fungi, and other microbial eukaryotes. Investigations utilizing diverse model systems have defined an active role of the microbiota in both normal host physiology (1) and disease (2, 3). Bacteria are the main components of the microbiota, having been shown to comprise >99% of the digestive tract microbial cohort (4). Bacteria are the best understood constituent of the microbiota, often serving diverse roles in host physiology (5). In recent years, the fungal component, or “mycobiota,” is becoming increasingly better recognized (6–8). It is estimated to constitute 0.01–0.1% of the microbial community in humans (4, 8, 9); however, the fungal contribution to the microbiome could be under-represent by the paucity of fungal genome reference sequences (8, 10). The gut mycobiome is not stable over time (7, 11, 12) and the low abundance and diversity of its composition are influenced by different factors, such as diet, gender, age, and geographical location (13, 14).

A recent report suggests that fungi do not efficiently colonize the digestive tract, rather their presence in the gut may be linked to both diet and the oral flora (15). However, a number of studies demonstrate that the presence of commensal fungi could be important for immune homeostasis. Fungal dysbiosis is associated with several disease etiologies (10, 16, 17). Interspecific interactions among fungi and fungi-bacteria and/or fungi/host can significantly impact health and disease (16). The gut mycobiome has been linked to different gastrointestinal pathologies, including: inflammatory bowel disease (IBD) (18, 19), irritable bowel syndrome (20, 21), peptic ulcers (22), antibiotic associated diarrhea (23) as well as graft-vs.-host disease (24, 25), and to crosstalk with the brain (26).

Although there is a dearth of knowledge on if and how fungi interact with host immune system components under normal “commensal” or healthy conditions or during times of microbial dysbiosis, much is known about how diverse innate immune receptors interact with fungal pathogens (10, 27). Innate immune cells, which have a phylogenetic history extending well-beyond the vertebrates, are equipped with a range of pattern recognition receptors (PRR) to sense and interact with different fungal pathogen-associated molecular patterns (PAMPs), as components of fungal cell wall, including β-glucan, mannans, mannoproteins, chitin, and also fungal-derived nucleic acids (10, 27). Notably, most of these PRRs are localized on the cell surface or in the cytoplasm of blood cells (17, 28) and the binding to their ligands shape antifungal immune responses, activating various signaling cascades that result in fungal internalization via phagocytosis, cytokine production, and/or production of reactive nitrogen and oxygen species (29, 30). Whereas, it has been shown that certain immune effectors influence the growth of bacteria in ways that often promote stable biofilms and/or shape barrier defenses (31–35), equivalent responses have yet to be documented for any fungal species.

We previously have described the variable region containing chitin binding proteins (VCBPs)-encoding gene families in two protochordate model systems (36–38). In *C. robusta*, four members of the VCBP gene family (VCBP-A, -B, -C, and -D) have been described (38). VCBP A-C consist of two immunoglobulin (Ig)-like variable domains at the N-terminus and a chitin binding domain (CBD) at the C-terminus (39). Unique temporal-spatial expression patterns among VCBP genes during morphogenesis, suggest a role in gut development that also may impact colonization dynamics (40). VCBP-C is the most extensively studied (34, 38). The Ig domains of VCBPs bind bacteria and promote phagocytosis or shape biofilm formation (34, 38); a physiological role for the CBD is less clear. We show here that the CBD of VCBP-C binds chitin molecules present on the cell walls of fungi isolated from the *Ciona* gut. VCBP-C may well-serve an integral role in promoting transkingdom, bacterial-host-fungal interactions within the gut microbiome, a heretofore-unrecognized function for an immunoglobulin-related molecule.

## Materials and Methods

### Isolation of Fungi From the *Ciona robusta* Gut

Upon arrival, wild-harvested animals were allowed to acclimate in 0.22 μm-filtered artificial seawater (ASW) for 3–4 h. All steps to isolate fungi were carried out under a laminar hood, in aseptic conditions, using sterile materials. Briefly, after sterilization of the *Ciona* tunic with 70% ethanol and washing with sterile ASW (41), gut was excised surgically (aseptically) from five animals and disrupted using a Dounce homogenizer to liberate bacteria and fungi from the mucosal surface of the organ. Host tissue was separated by processing through a 40 μm filter at 1200 × g for 10 min. Gut microorganisms were collected by pelleting at 500 × g or 10 min, washing and re-suspending in 1 ml ASW. An aliquot of this suspension (300 μl) was plated onto Yeast Peptone Dextrose (YPD) Agar plates (2% BactoPeptone, 1% Yeast extract, 2% dextrose, and 2% Agar) dissolved in ASW with Penicillin/Streptomycin (200 U/ml and 0.2 mg/ml, respectively; Fisher BP295950) antibiotics. Fungi were grown for 7–10 days before clonal growth was established and maintained by replica-streaking on YPD plates without antibiotics. Each fungal isolate was then inoculated in 5 ml YPD liquid medium, grown for 1 week in an orbital shaker at 20°C and then mixed with glycerol (10% final concentration) for long-term storage at −80°C.

Fungal species were identified by sequencing (Sanger) across the interspersed spacer (ITS) region of the 18s rRNA gene. Briefly, using Dneasy PowerSoil kit (Qiagen, Cat#12888), DNA extraction was performed on fungi grown in liquid medium for 5–7 days; fungal-specific18s rRNA amplicons were generated with standard ITS primers [ITS1 and ITS4, (42)]. Sequence data were deposited in GenBank and accession numbers are reported in Supplementary Table 1.

### Immunofluorescence of Cultured Fungal Isolates Cryo-Sectioned and Labeled With Fc-CBD-C Probe

Each fungal isolate was grown on a YPD agar plate for about 5 days and fixed with 4% paraformaldehyde in PBS overnight at 4°C. Plates were washed three times for 10 min in PBS and small pieces of colonies were embedded and frozen on dry ice in OCT freezing media (Fisher Scientific), and stored at −80°C. Thin sections (7 μm) were collected on poly-L lysine (MP Biomedicals, #0210269125)-coated slides. Immunofluorescence staining was performed using a recombinant, customized, probe possessing a N-terminal human Fc domain fused to the VCBP-C CBD domain, and named the Fc-CBD-C probe. The specificity of this probe for chitin, and the general staining procedures, were previously validated and described in Dishaw et al (34). Briefly, slides were washed twice with PBS and incubated first for 10 min with animal-free blocking solution (Vector Laboratories #SP-5030) diluted in PBS and then incubated with the Fc-CBD-C probe diluted 1:10 in blocking solution for 2 h at room temperature. Control staining experiments were run in parallel using only a human Fc probe. After three PBS washes, slides were incubated with goat anti-human Fc Alexa Fluor 488 antibody (Invitrogen, #H10120) diluted 1:1000 in PBS for 45 min and washed again twice with PBS. Cellular nuclei were stained with Hoechst (Invitrogen, #H3570) diluted 1:2000 in PBS for 20 min. After a PBS wash for 5 min, slides were mounted with Aquamount (Fisher #143905) and viewed using fluorescence microscopy. Overexposed images of control experiments were acquired to verify lack of specific signal compared to those from the experimental slides (fungi incubated with Fc-CBD-C probe), and to verify the presence/absence of diffuse background signal in the controls.

### Immunofluorescence Detection of Whole Mount Fungi With the Fc-CBD-C Probe

Fungal strains were grown in YPD liquid medium and orbital shaking at 20°C for 7 days, following which fungal bodies were fixed in 4% paraformaldehyde in PBS overnight at 4°C. Samples were washed several times in PBS and processed for whole mount immunofluorescence staining with the Fc-CBD-C probe as described above. Controls were run in parallel and images acquired as described in the previous section Immunofluorescence of cultured fungal isolates cryo-sectioned and labeled with Fc- CBD-C probe.

### Isolation of Fungal Spores for Immunofluorescence and VCBP-C Binding

Spores were isolated from fungal cultures grown for 7 days in 10 ml YPD liquid medium with orbital shaking at 20°C. Briefly, the culture was filtered using twice folded sterile gauze and centrifuged at 2200 × g for 15 min. Spores were re-suspended in 1 ml YPD medium and, either were fixed for immunofluorescence staining with the Fc-CBD-C probe or incubated with VCBP-C recombinant protein, as describe below.

For the CBD-C binding, spores were fixed by adding formamide (4% final concentration) to 400 μl of re-suspended spores, followed by incubation for 30 min with gentle agitation. Spores were pelleted at 2600 × g for 10 min, re-suspended in PBS and plated on poly-L-lysine coated circle glass coverslips (see above). The spore suspension was incubated for 15 min to allow attachment to the coverslip, then the immobilized spores were incubated overnight at 4°C with the Fc-CBD-C probe and BSA 0.5% in PBS was used as blocking solution before detection with secondary antibody.

In order to characterize the biding of VCBP-C, 300 μl of spores were incubated for 2 h with gentle agitation, either with 50 μg/ml VCBP-C recombinant protein (prepared as described in (34), or the same volume of 10 mM Tris pH 8/50 mM NaCl solution as a control. Spores were collected by centrifugation at 2600 × g for 10 min to remove unbound VCBP-C protein, re-suspended in 500 μl PBS, fixed and plated on poly-L lysine coated coverslips as described above. Binding of VCBP protein to spores was detected by immunofluorescence using an anti-VCBP-C antibody (34, 38, 40). Chitin molecules on spore cell walls also were stained with wheat germ agglutinin (WGA) Texas-Red conjugate (Invitrogen, # W7024) diluted 1:200 in PBS and incubated for 20 min. WGA solution was removed with two PBS washes and then nuclei were stained with Hoechst and mounted as described previously.

## Results

### Chitin-Binding Domain of VCBP-C Recognizes Chitin Molecules in Fungal Cell Wall

In order to define the *Ciona* gut mycobiome, fungi were recovered from the gut of *C. robusta* using culture-based isolation methods. The isolates recovered in this study include eight different *Penicillium* spp. and five different *Trichoderma* spp., identified by ITS sequencing. Other filamentous fungal species that were recovered include: *Acremonium* sp., *Acrostalagmus* sp., *Arthrinium* sp., *Mucor* sp., *Phaeosphaeria* sp., and *Phoma* sp. It is essential to recognize that the approaches used facilitated the isolation and identification of only those fungal species that could be cultured in the conditions defined in this study; hypothetically, these may represent only a small proportion of the *Ciona* gut mycobiota.

**Figure 1:**
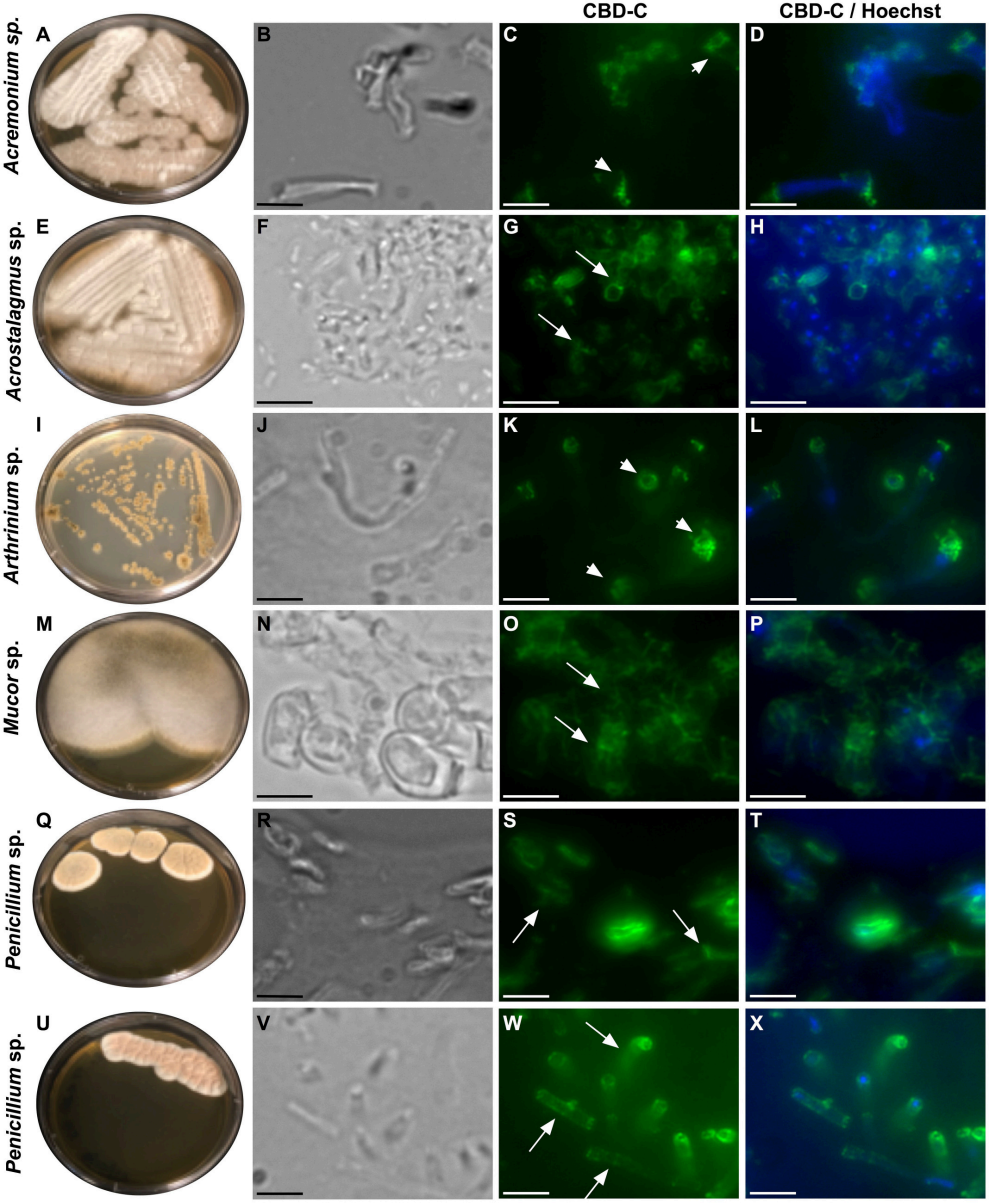
Immunofluorescence with the Fc-CBD-C probe on fungal sections. Several species of fungi, including: *Acremonium* sp., *Acrostalagmus* sp., *Arthrinium* sp., *Mucor* sp., and *Penicillium* spp., were isolated from *Ciona* gut and grown on YPD agar plates **(A,E,I,M,Q,U)**. Immunofluorescence staining, using the Fc-CBD-C probe and detected with a fluorescent secondary antibody (Alexa Fluor 488), on OCT-embedded and cryo-sectioned fungal isolates, indicates that chitin molecules are distributed in the whole cell wall of most of the fungi analyzed (**G,O,S,W**, arrows). In two fungal species, the Fc-CBD-C probe detects chitin localized in specific regions of the fungal cell wall (**C,K**, arrowhead). **(B,F,J,N,R,V)** are brightfield images of fungal sections; **(D,H,L,P,T,X)** represents merged images of the Fc-CBD-C probe (green) and nuclear staining with Hoechst (blue). Scale bars: **(B–D,J–L,R-T,V–X)**, 5 μm; **(F–H, N–P)** 10 μm.

**Figure 2:**
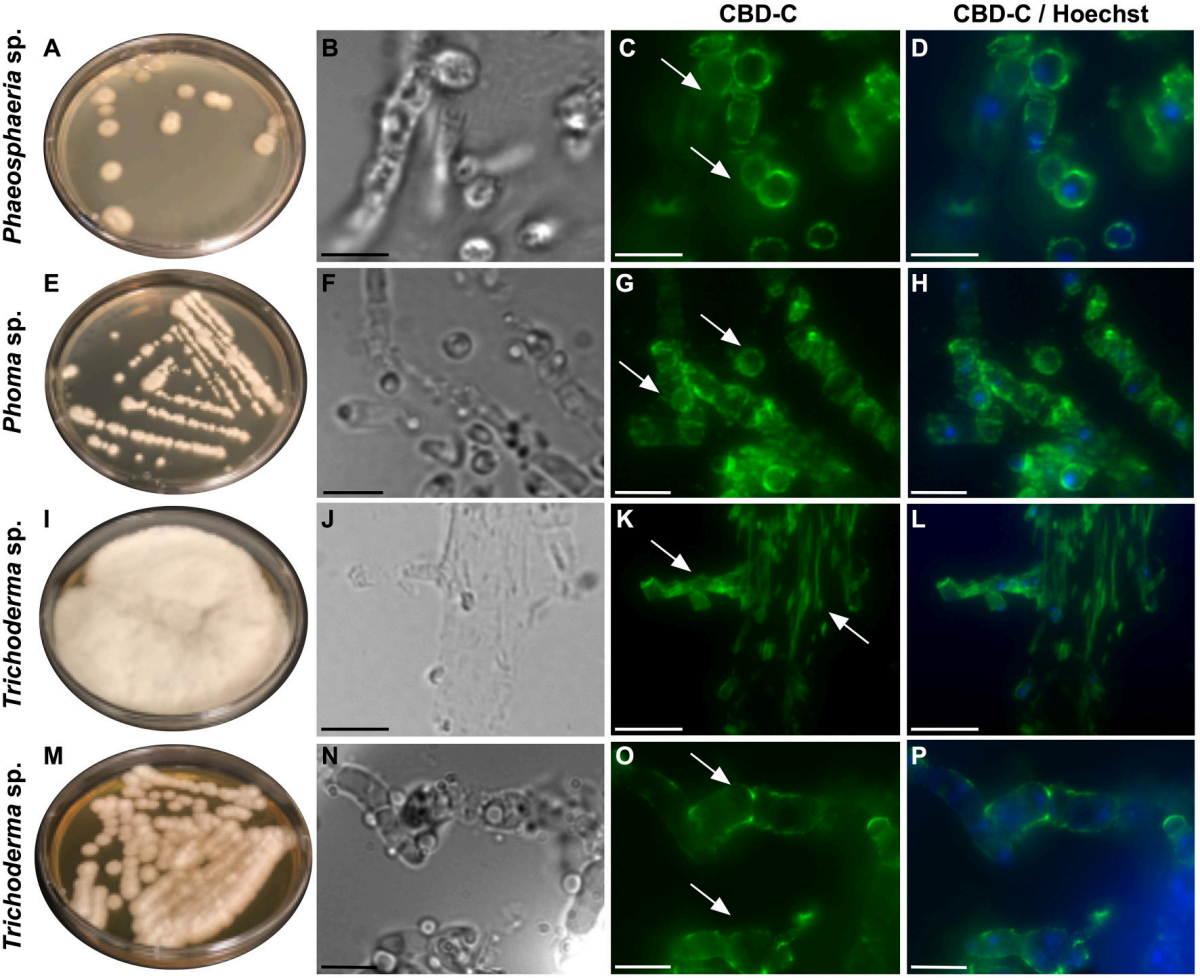
Immunofluorescence with the Fc-CBD-C probe on fungal sections. Fungal species, including *Phaeosphaeria* sp., *Phoma* sp., and *Trichoderma* spp., were isolated from the *Ciona* gut and grown on YPD agar plates **(A,E,I,M)**. Immunofluorescent staining, using the Fc-CBD-C probe, on OCT-embedded and cryo-sectioned fungal isolates, indicates that chitin molecules are distributed in the whole cell walls of these fungi (**C,G,K,O**, arrows). **(B,F,J,N)** are brightfield images of fungal sections; **(D,H,L,P)** represent merged images of the Fc-CBD-C probe (green) and nuclear staining with Hoechst (blue). Scale bars: **(B–D,J–L)**, 10 μm; **(F–H,N–P)**, 5 μm.

**Figure 3:**
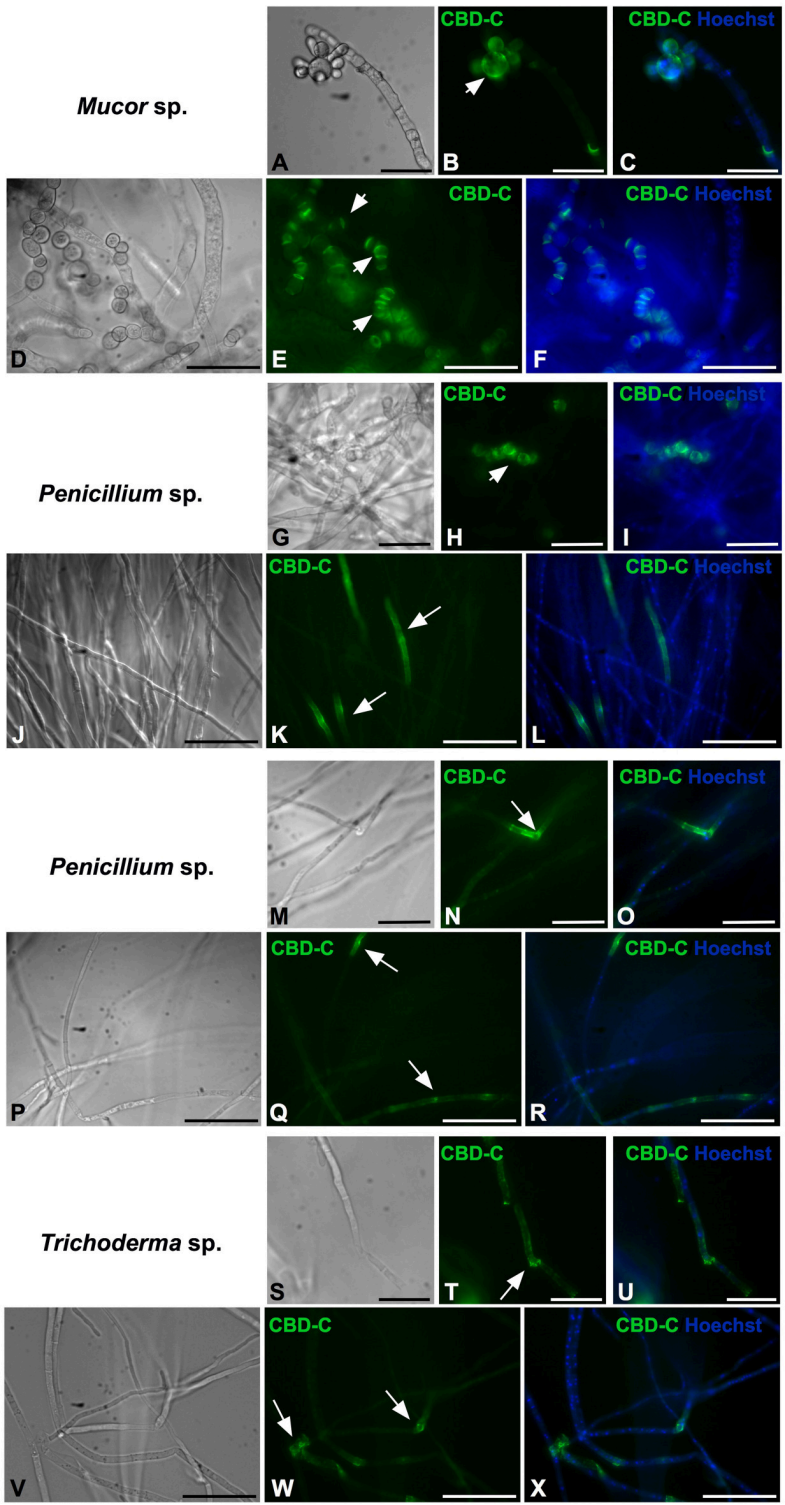
Immunofluorescence with the Fc-CBD-C probe on whole fungi grown in liquid medium. Fungal species, *Mucor* sp. **(A–F)**, *Penicillium* spp. **(G–R)**, and *Trichoderma* sp. **(S–X)**, were grown in liquid medium and characterized by immunofluorescent staining with the Fc-CBD-C probe. In *Mucor* sp., CBD-C binds fungal spores (**B,E**, arrowheads); in *Penicillium* spp., CBD-C recognizes chitin in specific regions of the hyphae (**K,N,Q**, arrows) and in some cases fungal spores (**H**, arrowhead); in *Trichoderma* sp., chitin fibers are apparent in specific regions of the hyphae (**T,W**, arrows). **(A,D,G,J,M,P,S,V)** represent brightfield images; **(C,F,I,L,O,R,U,X)** represents merged images of the Fc-CBD-C probe (green) and nuclear staining with Hoechst (blue). Scale bars: **(A–C,D–F,J–L,P–R,V–X)**, 50 μm; **(G–I,M–O,S–U)**, 25 μm.

All of the isolates were cryo-sectioned and stained with the Fc-CBD-C probe. Chitin fibers were localized in the cell wall of the fungal hyphae (Figures 1, 2), whereas control experiments performed using only the human Fc-probe do not show any fluorescent signal (Supplementary Figure 1). In almost all the fungal isolates, CBD-C binds the whole cell wall (Figures 1G,O,S,W, 2); however, in *Acremonium* sp. (Figure 1C) and *Arthrinium* sp. (Figure 1K), binding is localized predominantly along the edges of the cell wall. The observed staining patterns do not demonstrate if the CBD is detecting chitin in the inner or outer layer of the cell wall, an important distinction in characterizing the overall function of VCBP molecules. In order to resolve this distinction, whole mount staining with the Fc-CBD-C probe was carried out with *Mucor* sp., *Penicillium* spp., and *Trichoderma* sp. grown in liquid medium. In *Mucor* sp., the CBD-C mainly binds the spores that are produced in great abundance, but it does not bind the fungal hyphae (Figures 3B,E). In the two *Penicillium* spp. and in the *Trichoderma* sp., the CBD-C staining is highly regionalized along fungal hyphae (Figures 3K,N,Q,T,W). In one of the *Penicillium* sp., spores are bound by CBD-C, as observed in *Mucor* sp. (Figure 3H). Control experiments using the human Fc-probe show no specific binding to the whole fungal bodies (Supplementary Figure S2).

**Figure 4:**
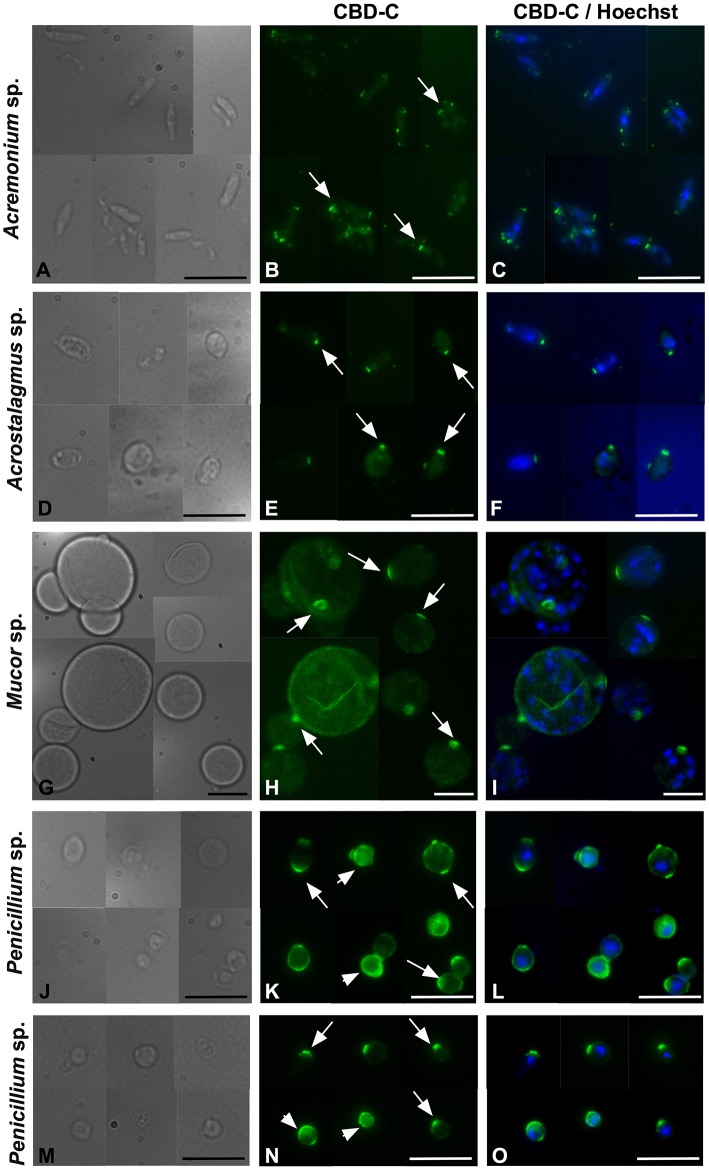
Immunofluorescence with the Fc-CBD-C probe on fungal spores. In *Acremonium* sp., *Acrostalagmus* sp., and *Mucor* sp. the CBD-C probe detects chitin fibers in regionalized areas on the surface of spores isolated from liquid cultures (**B,E,H**, arrows). In *Penicililum* spp., CBD-C binds almost the entire spore wall (**K,N**, arrowheads) in most spores. In some spores, chitin is instead detected in localized spots (**K,N**, arrow). **(A,D,G,J,M)** represents brightfield images; **(C,F,I,L,O)** represent merged images of the Fc-CBD-C probe (green) and nuclear staining with Hoechst (blue). Scale bars: 10 μm.

To further investigate the binding of CBD-C to fungal spores, *Acremonium* sp. *Acrostalagmus* sp., *Mucor* sp., and *Penicillium* spp., were grown in liquid medium and spores were isolated. The Fc-CBD-C probe binds the surface of spores recovered from all of these fungal species (Figure 4). In *Acremonium* sp. *Acrostalagmus* sp., and *Mucor* sp., CBD-C exhibits regionalized binding on the spore surface (Figures 4B,E,H). In the *Penicillium* spp., CBD-C binds almost the entire spore wall; however, in some spores, chitin binding is localized to discrete regions (Figures 4K,N). One explanation for the observed differences in localization of chitin is that it may reflect different stages of the spore cell cycle. No staining was observed in control experiments (Supplementary Figure 3).

Collectively, these results demonstrate that the CBD of VCBP-C binds chitin that is localized mainly on the surface of fungal spores. In parallel studies, the dye-conjugated plant lectin, wheat germ agglutinin (WGA), was used to confirm some of these observations; specifically, WGA recognizes chitin fibers localized only on fungal surfaces and not in the inner cell walls (43, 44); hence, this lectin was utilized, as described in the following section, to verify the presence of chitin on fungal surfaces and to confirm the degree of overlap with signals detected by the CBD of VCBP-C. Moreover, chitin molecules that are recognized by CBD-C were not found to be exposed throughout the entirety of the cell walls of fungi, rather are localized to specific regions, e.g., distinct segments of hyphae.

### VCBP-C Binds Chitin Exposed on the Surface of Fungal Spores

**Figure 5:**
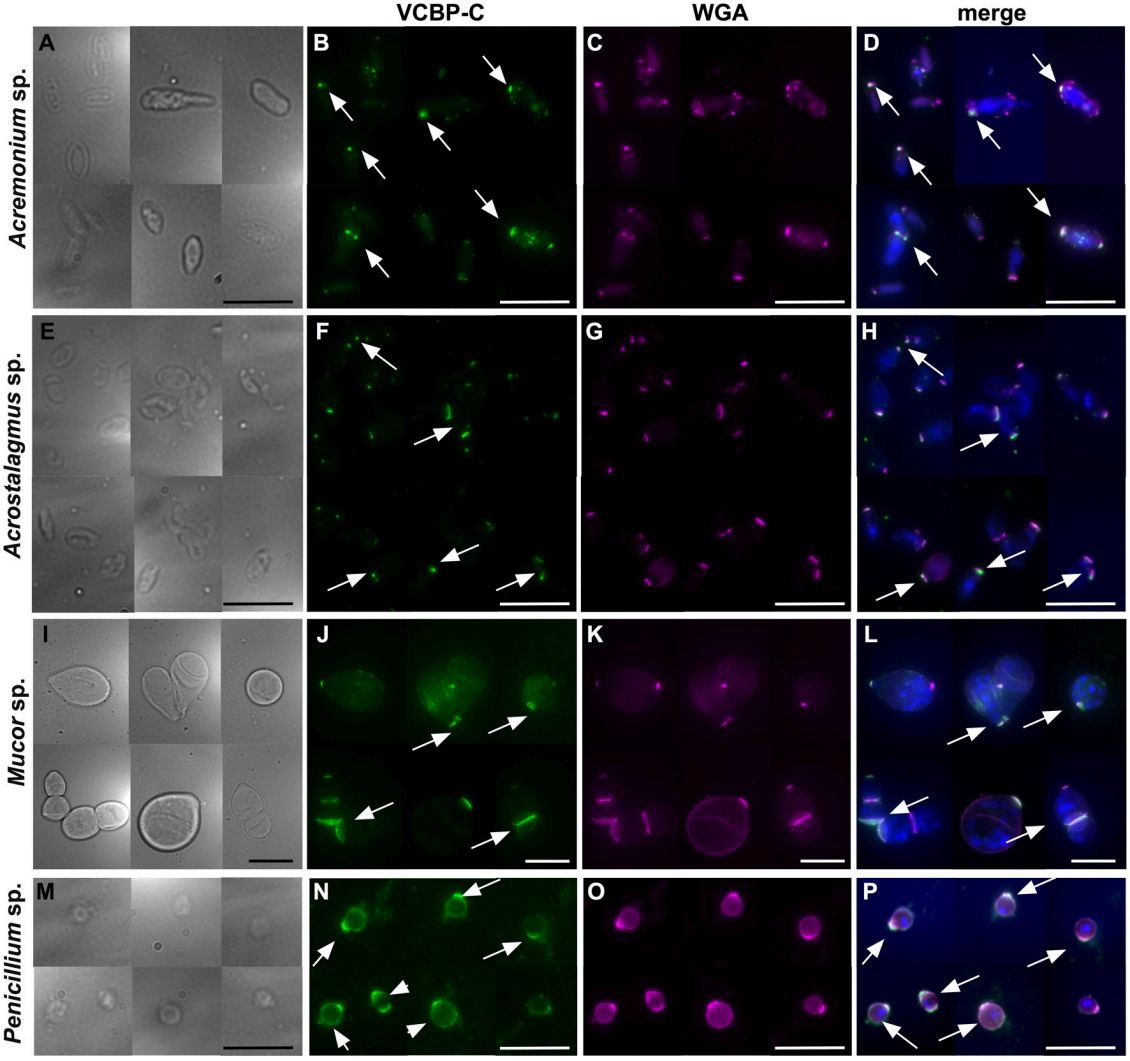
Immunofluorescence on fungal spores incubated with VCBP-C protein and WGA. VCBP-C protein incubated with spores isolated from liquid cultures of *Acremonium* sp., *Acrostalagmus* sp. and *Mucor* sp. binds chitin localized in specific regions of the spore surface (**B,F,J**, arrow). In *Penicillium* sp., VCBP-C recognizes chitin surrounding the entire spore (**N**, arrowheads) or in some cases, localized to specific spots (**N**, arrow). WGA staining **(C,G,K,O)** confirms the presence of chitin fibers on fungal spores. WGA co-localizes with VCBP-C staining in almost all of the spores, as observed in the merged images (**D,H,L,P**, arrows). **(A,E,I,M)** represent brightfield images; **(D,H,L,P)** represent merged images: VCBP-C in green, WGA in magenta and nuclear staining with Hoechst in blue. Scale bars: 10 μm.

In order to determine if the full-length VCBP-C exhibits the same binding pattern to fungi that is exhibited by its CBD (in the form of the Fc-CBD probe), *Acremonium* sp. *Acrostalagmus* sp., *Mucor* sp., and *Penicillium* sp., were grown in liquid medium, processed for isolation of spores and incubated with recombinant VCBP-C protein. Intact VCBP-C exhibits the same binding pattern to the cell wall surface of these species (Figure 5) that was observed with the CBD probe (Figure 4), whereas no staining is observed in control samples (Supplementary Figure S3). Specifically, in *Acremonium* sp. *Acrostalagmus* sp., and *Mucor* sp., VCBP-C recognizes chitin localized in specific regions of the spore surface (Figures 5B,F,J); in *Penicillium* sp., the staining either surrounds the entire spore or is detected in specific regions (Figure 5N). WGA lectin was also used to stain the same isolates of spores that were incubated with the VCBP-C protein (Figures 5C,G,K,O) and the fluorescent signals co-localize (Figures 5D,H,L,P), further confirming the specificity of VCBP-C binding to chitin molecules that are localized on fungal surface.

## Discussion

*Ciona robusta* is a model organism that in recent years has been adapted to studies of host-microbe interactions within the gut (45, 46), and has introduced an entirely unique, non-vertebrate model system to complement studies performed in mouse and zebrafish models (46). Disparate populations of *Ciona* share a core microbiome of distinct bacterial taxa (47). More recently we have shown that the gut virome of *Ciona* is temporally stable and is dominated by prophages (48). The isolation of diverse microorganisms from the *Ciona* gut, including bacterial strains [(34) Dishaw et al., unpublished data], different phages (49, 50) and, as described here, fungal species, is a critical first step in allowing us to investigate the underlying mechanisms that govern the bacterial, viral, and fungal relationships that define homeostasis in the gut of an organism relying solely on innate immunity (51). We have shown previously that epithelial barriers and associated innate functions are phylogenetically ancient and have evolved different mechanisms to maintain dialogue with adherent microbiota (46). However, such relationships appear to have been preserved and are remarkably insightful in gaining a better appreciation of the nature and dynamics of prokaryotic-eukaryotic relationships.

Here, we show that a VCBP, through its CBD, binds chitin localized in the cell wall of filamentous fungi isolated from the gut of *Ciona*. In most fungi, i.e., *Candida* yeasts, *Saccharomyces cerevisiae, Pneumocystis* spp., *Aspergillus* conidium, *Aspergillus fumigatus* hyphae, *Cryptococcus, Histoplasma capsulatum*, and *Blastomyces dermatitidis* (52), chitin polymers, together with β-1,3- and β-1,6 glucans, are localized within the inner layer of the fungal cell wall where they form a basket-like scaffold around the cell (52–54). The integrity of this scaffold is important for the fungal cell to ensure plasticity, allowing turgor-driven cell expansion as well as robustness and to prevent bursting of the cell (52). Although VCBP-C recognizes chitin within the fungal cell wall, the binding patterns to the whole fungal body indicate that at least some of the chitin is exposed on the fungal cell surface. Binding of the CBD-C probe to *Mucor* sp. spores and some regions of the *Penicillium* spp. and *Thricoderma* sp. hyphae (Figure 3), suggest that these regions could be undergoing cell wall reorganization for production of spores or for hyphal growth in the intercalary compartments. Although the hyphal extension in filamentous fungi normally is confined to the extreme apex, there are exceptions where this process is observed in the intercalary regions of fungal hyphae (55). In yeast cells, bud scars, which are located on the surface of the mother cell and mark the site of cytokinesis and septation from daughter cell, have fewer outer cell wall layers, thereby exposing the inner chitin-glucan to the external environment (56). VCBP-C recognizes chitin fibers on spores isolated from fungal species, such as *Acremonium* sp., *Acrostalagmus* sp., *Mucor* sp., and *Penicillium* spp. (Figures 4, 5). Different patterns of localization, reminiscent of bud scars are evident. Collectively, the findings are consistent with VCBP-C binding fungal spores or those structures that could originate spores, which in *Ciona* could be ingested through filtration of water and reside in the gut.

In vertebrates, chitin is recognized by the murine mannose receptor, Nod2, and TLR9 (57). Although chitin is localized within the inner layers of fungal cell walls and typically is not exposed to innate immune cells, it is recognized as an important immune-reactive polysaccharide (17). Ultrapurified chitin from *C. albicans* blocks the recognition of this yeast by human peripheral blood mononuclear cells (58). Chitin purified from *Aspergillus* induces the production of IL-1Ra, an anti-inflammatory cytokine and also stimulates proinflammatory mechanisms (i.e., IL-1β production) when it is present in combination with costimulatory PAMPs (59). The immune response to chitin molecules is complicated by the finding that different particle sizes are recognized by a different combination of PRRs, activating distinct signaling pathways that consequently induce the secretion of diverse cytokines (60, 61). Recently, a role for chitin also has been suggested in trained immunity (62), a form of immunological memory exhibited by innate immunity (63). Whereas, the downstream effects of VCBP-C and chitin interactions, specifically on living fungal species, may involve the shaping of activation-inhibition of developmental pathways in fungal cells, a role for VCBPs in regulating immune responses that shape fungal colonization of the gastrointestinal tract also may be possible. Future studies may help to decipher mechanisms used by the host recognition of fungi within the gut that may have phylogenetic significance.

The capacity of VCBP-C to bind both bacteria and fungi, suggests that the *Ciona* gut may represent unique challenges for establishing and maintaining homeostasis in this high flow rate, filter feeder. Through interactions involving the IgV domains, VCBP-C binding enhances bacterial phagocytosis by *Ciona* immunocytes (38) and modulates biofilm formation among bacteria isolated from the *Ciona* gut (34). The studies reported here indicate that CBD-C binds fungi by recognizing cell surface-exposed chitin molecules. Furthermore, the location of chitin detected by the CBD probe likely reflects physiologic or developmental states of the fungi. It is attractive to consider that the recognition of both bacteria and fungi may in some way facilitate protection against pathogens or support the colonization of gut symbionts; however, the dynamics likely are complicated. Specifically, in *Ciona*, the endogenous expression of chitin-rich mucus coating the gut epithelium has been shown previously to capture and bind VCBPs via the CBD (34). Thus, VCBPs can be free in the lumen as well as tethered to the chitin-rich mucus. Furthermore, the production of chitinases by some colonizing bacteria could release surface-trapped VCBPs, thereby facilitating recognition and interactions with chitin-rich fungal structures.

Although this may represent an expansive interpretation of the relationship of VCBPs to bacterial and fungal components of the gut, it is well-recognized that fungal and bacterial communities in the gut influence each other, through both positive and negative associations (7, 16, 19, 64). A transkingdom association between potentially pathogenic bacteria, as *E. coli* and *Serratia marcescens*, and yeast as *C. tropicalis* has been described in Crohn's disease (65). Fungal-bacterial interactions also have been demonstrated in an animal model where *Saccharomyces boulardii* interacts with and alters commensal bacteria, alleviating acute liver failure (66). Hence, bacteria and fungi may regulate each other in the commensal community using different mechanisms, that only now are being recognized and may offer novel targets for manipulating the microbiome (64). The novelty of the studies reported here is that this process may be modulated by the host and mediated by a bifunctional immune receptor.

The VCBP gene family is unique to protochordates (36–38), and thus the relationships that we propose may be unique among molecular mediators. However, the VCBPs may share some functional similarities to other classes of immune molecules found in vertebrates, such as IgA, that often exert their effects via immune inclusion-exclusion mechanisms (34, 40, 46, 67, 68). Most studies focus on the colonization of microbiomes by bacterial communities; however, in regards to colonization by fungi, there have only been a few studies demonstrating a protective and/or regulatory role for secreted immune mediators such as IgA against, for example, systemic candidiasis (69–71). Here, the unique binding of VCBP-C to both bacteria and fungi may influence the positive and negative crosstalk among resident microorganisms and consequently influence the dynamics of co-colonization and the establishment and maintenance of the gut microbiota, providing a first example of transkingdom interactions modulated by secreted immune mediators.

## Ethics Statement

The research described herein was performed on *C. robusta* a marine invertebrate collected in Mission Bay near San Diego, CA (M-REP, Carlsbad, CA, USA), in locations that are not privately-owned nor protected in any way. *Ciona* is considered an invasive species and is not regulated or protected by environmental agencies in the United States. The collection service contracted in this study maintains current permits and licenses for collection and distribution of marine invertebrates to academic institutions. Handling of live animals was in accordance with the guidelines of our academic institutions. Animals were recovered and brought to the laboratory alive and maintained in clean water with aeration. In accordance with general animal protocols, the least number of animals required per experiment were utilized. Animal waste products were disposed of in accordance with USF Health and Safety Guidelines.

## Data Access

The sequences reported in this manuscript have been deposited in the Genbank database with the following accession numbers: MK431096, MK424129, MK424120, MK423932, MK424124, MK424125, MK424126, MK424132, MK424131, MK424130, as reported more in detail in the Supplementary Table 1.

## Authors Contributions

AL designed, performed, and analyzed experiments and wrote/edited the manuscript. JC and GL edited the manuscript. LD conceived the original idea and wrote/edited the manuscript.

### Conflict of Interest Statement

The authors declare that the research was conducted in the absence of any commercial or financial relationships that could be construed as a potential conflict of interest.
